# High Electron Mobility in Epitaxial Trilayer Graphene on Off-axis SiC(0001)

**DOI:** 10.1038/srep18791

**Published:** 2016-01-07

**Authors:** Mahdi Hajlaoui, Haikel Sediri, Debora Pierucci, Hugo Henck, Thanyanan Phuphachong, Mathieu G. Silly, Louis-Anne de Vaulchier, Fausto Sirotti, Yves Guldner, Rachid Belkhou, Abdelkarim Ouerghi

**Affiliations:** 1 CNRS- Laboratoire de Photonique et de Nanostructures, Route de Nozay, 91460 Marcoussis, France; 2 Synchrotron-SOLEIL, Saint-Aubin, BP48, F91192 Gif sur Yvette Cedex, France; 3 Laboratoire Pierre Aigrain, Ecole Normale Supérieure-PSL Research University, CNRS, Université Pierre & Marie Curie-Sorbonne Universités, 24 rue Lhomond, 75005 Paris, France

## Abstract

The van de Waals heterostructure formed by an epitaxial trilayer graphene is of particular interest due to its unique tunable electronic band structure and stacking sequence. However, to date, there has been a lack in the fundamental understanding of the electronic properties of epitaxial trilayer graphene. Here, we investigate the electronic properties of large-area epitaxial trilayer graphene on a 4° off-axis SiC(0001) substrate. Micro-Raman mappings and atomic force microscopy (AFM) confirmed predominantly trilayer on the sample obtained under optimized conditions. We used angle-resolved photoemission spectroscopy (ARPES) and Density Functional Theory (DFT) calculations to study in detail the structure of valence electronic states, in particular the dispersion of π bands in reciprocal space and the exact determination of the number of graphene layers. Using far-infrared magneto-transmission (FIR-MT), we demonstrate, that the electron cyclotron resonance (CR) occurs between Landau levels with a (B)^1/2^ dependence. The CR line-width is consistent with a high Dirac fermions mobility of ~3000 cm^2^·V^−1^·s^−1^ at 4 K.

Monolayer graphene is a two-dimensional materials with linear dispersions near the *K* and *K*’ points of the Brillouin zone. Low-energy charge carriers therein obey the Dirac-Weyl equation and behave like massless fermions^1^. This material is ambipolar and exhibits ballistic transport properties on a micrometer scale up to room temperature. In multilayer graphene, the interlayer coupling introduces perturbations of the low-energy band dispersions^2^. Consequently, the linear *π* and π* bands near the Fermi level in monolayer graphene are modified in multilayer graphene, showing a strong dependence on the stacking sequence as well as the layer number. Recently multilayer graphene has attracted considerable interest^3^ due to the possibility of inducing a band gap. This band gap can be generated by breaking the translational symmetry using an external electric field^4^,^5^ or by introducing asymmetrical doping between the graphene layers resulting in a ‘built in’ electric field^3^,^6^. Moreover, the number of graphene layers and their stacking configuration will control the electronic properties of multilayer graphene. When multilayer graphene are stacked, Bernal stacking represents the lowest energy configuration. The shift of one atom of the bottom layer along the C-C bond axis (one bond length) allows a transition between the Bernal and the second stable allotropes of multilayer graphene, rhombohedral stacking. In the case of trilayer graphene, this transition has to overcome, an energy barrier of about 1.1 meV/atom^7^.

A viable method for obtaining a large-scale graphene production is the epitaxial approach based on SiC graphitization. The epitaxial growth of graphene on semi-insulating SiC has the potential to enable the next generation of ultra high frequency and low power electronic devices. The main advantage here is the possibility of obtaining a large graphene area directly on semi-insulating substrates which is one of the main requirements for high frequency devices, with a huge control of the number of graphene layers. Moreover, the choice of the appropriate SiC polytype also makes it possible to select for the formation of Bernal or rhombohedral multilayer graphene^8^. Other methods to obtain large-area graphene include chemical vapor deposition (CVD) on metals^9^. However, this CVD on metal process is less suitable for device manufacture, as the graphene layers must be transferred onto a semiconducting substrate. These transferred graphene layers tend to acquire contamination as a result of the transfer process, thus resulting in graphene which is not suitable for device oriented applications. Coletti *et al.*^10^, studied epitaxial trilayer graphene obtained using hydrogen intercalated bilayer graphene on 3C-SiC(111) and on 6H-SiC(0001) substrates using ARPES. Their ARPES results suggest that the resulting trilayer graphene showed a tendency towards the development of presumably large-area ABC and ABA type stacking respectively. Recently, Lalmi *et al.*^11^ studied trilayer graphene on 4H-SiC(0001) using STM/STS and ARPES. They showed the presence of a different types of structural defect^11^ in which a transition from ABA to ABC stacking was observed.

The Si-face 4 H-SiC substrate is preferred to the C-face for the growth of graphene. This is mainly due to the fact that graphene growth is easier to control on the Si-face, and under optimized conditions the thickness of the graphene can be limited to just a mono, bi and few layers over large areas. The off-axis 4H-SiC(0001) substrate could therefore, be a good candidate to obtain trilayer graphene. Although further, experiments are required to obtain large-area and high quality sample with high electron mobility.

We demonstrate here the successful growth of ordered trilayer graphene layers on off-axis SiC(0001) substrates up to an unprecedentedly large area of hundreds of micrometers. The high quality of the graphene layers was further confirmed by AFM, micro-Raman mappings and X-ray photoelectron spectroscopy (XPS) measurements. Using FIR-MT measurements, we show that trilayer graphene exhibits high electron mobility (~3000 cm^2^·V^−1^·s^−1^ at 4 K) for the observation of the electron cyclotron resonance, despite their high doping level. By comparing ARPES measurements and DFT calculations, we show that the ordered trilayer graphene presents an ABA stacking. Using the near-edge X-ray absorption fine structure (NEXAFS) technique, we also measured the unoccupied electronic states (π* and σ*). These observations could be of prime importance, particularly regarding the development of multilayer graphene-based electronic devices.

The trilayer graphene used in this study was obtained by annealing 4° off-axis 4H-SiC(0001) at 1550 °C in 800 mbar argon for 10 min (Fig. 1(a)). For Si terminated SiC substrates the first carbon layer which is formed, the so-called interface or buffer layer, is insulating because one third of its carbon atoms are covalently bound to the SiC substrate. The epitaxial graphene refers so to the carbon layer formed on top of this interfacial layer, and features linear dispersion typical of isolated single layer graphene. The thickness of our sample was confirmed by XPS and ARPES measurements. The typical morphology of the graphene sample can be seen in the AFM image displayed in Fig. 1(b). Step direction and terrace width are directly determined by the initial misorientation of the substrate with respect to the crystallographic (0001) plane. The (0001) terraces are characterized by a width of ~2–3 μm separated by steps heights of 10 nm, while the (11–20) nanofacets are characterized by a width of ~300–600 nm.

During the graphitization process, graphene growth starts in the vicinity of the nanofacets regions consisting of step edges^12^ and gradually propagates at the center of the terraces. This growth process generates a large area of carpet-like graphene, which covers the steps without interruption as clearly shown in the AFM image in Fig. 1(c).

In addition to AFM imaging, we carried out micro-Raman spectroscopy mapping. Figure 2(a,b) show the maps of the 2D peak position and *fwhm* on a 12 × 12 μm^2^ scan. The different color scale highlights a blue shift of the 2D peak from the terraces to the nanofacets, combined with an increase in the *fwhm*. This effect was not surprising, as the reduced bonding coordination of the nanofacets led to an easier Si sublimation from the substrate, which as explained before, facilitated graphene growth. This different growth rate between the terraces and the nanofacets may induce the formation of up to a trilayer along the nanofacets^6^. Taking two representative spectra from the terraces and nanofacets (Fig. 2(c)) it was easier to evaluate the shift and the *fwhm* of the 2D peak. As usual for epitaxial graphene on SiC, several intense peaks were visible in the wavenumber range between 1300–800 cm^−1^, corresponding to second-order Raman bands originating in the SiC substrate^13^. In addition to the 2D peak, other main structures originating from graphene were present: the D and the G bands. The low intensity of the D peak shows that there were only a small number of defects or disorders in the graphene structure^13^,^14^.

This is an indication of the high quality of epitaxial trilayer graphene on off-axis SiC under argon flux. As shown by the Raman maps, the 2D peak from the (11–20) nanofacets had blue-shifted with respect to those of the spectrum obtained for the (0001) terrace of about 50 cm^−1^ (from 2700 to 2750 cm^−1^), with an increased *fwhm* of about 60 cm^−1^ (from 50 to 110 cm^−1^). At the same time, we also saw a shift in the G peak of about 20 cm^−1^ (from 1590 to 1610 cm^−1^). This shift shows a strong correlation between the strain and the charge density on epitaxial graphene. Thus, the observed blue-shift indicates that there is a difference in strain (associated with the shift of the G and the 2D lines) and carrier density (associated with the shift of the G line) between the (0001) terraces and (11–20) nanofacets.

We probed the electronic properties of the sample further using XPS and NEXAFS experiments. The C1s spectra (Fig. 3(a)) were acquired using two different photon energies (hν = 340 eV, and hν = 510 eV). The variation in photon energy reflects a variation of surface and bulk sensitivity, allowing the depth positions of the different species to be identified across the surface. The different components contributing to the spectra were decomposed by a curve fitting procedure. The experimental data points are displayed with dots, meanwhile the solid line is the envelope of the fitted components. The C1*s* spectra show the conventional deconvolution expected for epitaxial graphene on SiC(0001)^12^,^15^,^16^, characterized by three contributions. The peak G, at a binding energy of 284.6 eV, indicates the presence of sp^2^ hybridized C–C bonds and it this is the signature of the graphene layers. This component was fitted using a Doniach-Sunjic line shape with an asymmetry factor α of 0.1 and a *fwhm* of 0.5 eV. The two other peaks at binding energies of 283.8 eV (SiC) and 285.3 eV (IL) are attributed to the SiC bulk and to the interfacial layer respectively. Due to the non-metallic nature of these components, the peaks are symmetric (asymmetric factor α = 0 eV). Assuming that the graphene-SiC sample can be modeled as a semi-infinite SiC substrate with a uniform graphene overlayer, the graphene thickness can be calculated from the ratio between the intensity of the G and SiC components^17^ extracted from XPS data using the following relation:


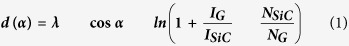


For a photon energy of 510 eV, we have an escape depth λ ~ 7.2 Å. In the geometry of our XPS experiment the take-off angle α was 0°, and 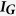 and 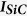 represented the area of the G and SiC components of the C 1s peak extracted from the deconvolution of Fig. 3(a – top). For epitaxial graphene on SiC, the areal density of the C atoms in graphene 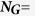
3.8 × 10^15^ cm^−2^ is about three times of that of the C atoms in the SiC bilayer 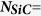 1.22 × 10^15^ cm^−2^. We obtained a thickness 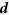 = 7.9 Å, corresponding to about three layers of graphene.

Unoccupied electronic states of the trilayer graphene were also examined by NEXAFS spectroscopy. The C k-edge spectra were measured for different incidence angles of linear polarized synchrotron light. As shown in the insert of Fig. 3(b), θ is the angle between the sample surface and the incident photons. The spectra present the characteristic features of epitaxial graphene on SiC^12^,^18^: two sharp resonances at 285.3 eV and 291.7 eV and a broad structure at 292.7 eV, assigned to the 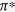, 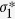 and 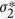 resonances, respectively. Changing the incidence angle of the light from grazing incidence (θ = 10°) to normal (θ = 85°) provided information on the orientation of the trilayer graphene with respect to the SiC substrate. As expected for a well-ordered planar π conjugated graphene system, the intensity of 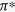 resonance was enhanced for grazing light incidence, when the direction of polarization vector 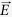 was close to the surface normal, and decreased with increases in the incident angle such that it wasalmost suppressed for normal incidence. Meanwhile the 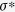 resonances revealed the opposite angular dependence behaviour. As no other significant features, apart from the σ* and π* transitions, were observed on these NEXAFS spectra, this meant the graphene layers presented a high degree of crystallinity and were uniformly parallel to the substrate surface. Moreover, the sharpness of the NEXAFS features and of the graphene C 1s core level peak a well-defined bonding environment and a long-range periodic order in the electronic structure of trilayer graphene.

The electronic structure was also probed using ARPES, which is a powerful method for this purpose, as it gives direct access to the spectral function containing the information on electron energy band dispersion. Figure 4(a) shows the valence band structure around the K point, along the MΓK direction, in the first graphene Brillouin zone. ARPES data are measured for hν = 60 eV. The momentum distribution curve (MDC) is shown in Fig. 4(b). The MDC was extracted for E – E_F _= –1.32 eV. The three peaks observed in the MDC result from the presence of three bands in the ARPES spectrum in Fig. 4(a). As is already well-known, multilayer graphene presents a band structure different from that of a single layer^19^. Furthermore, the band structure of few-layer graphene films depends on the number of layers, as well as on the stacking sequence. Clearer evidence of the band structure of our epitaxial graphene is given by the second derivative of the ARPES intensity map shown in Fig. 4(c). We observed the dispersion of three bands, as indicated by the MCD; two of these touching each other at the K point. These band dispersions showed evidence of a trilayer graphene on the off-axis 4H SiC (0001). They are plotted on Fig. 4(d) to give a comparison with theoretical data. This band dispersion was also consistent with our DFT calculations of ABA stacking. The ARPES measurements show the growth of Bernal stacked trilayer graphene. In particular, the sharp and intense structure of the three π-bands underlines the high structural quality of these graphene layers. However, we do not currently exclude the formation of the ABC stacking at low scale. As explained before, Bernal-stacking is energetically more favorable than rhombohedral stacking^7^,^20^, a fact that is confirmed by our results, where no contribution of ABC stacking was observed with respect to ABA stacking. As it is usually observed in the case of trilayer graphene grown on SiC, the π bands form a cone, for which the π branches cross at the Dirac point (E_D_) at –320 meV below the Fermi level (E_F_). This energy shift ΔE (ΔE = E_D_ − E_F_) is known to be related to the charge transfer from the substrate to the graphene layers^21^. From the position of the Dirac point,with respect to the Fermi energy, we estimate a carrier concentration *n* ~ 9 × 10^12 ^cm^−2^. Considering that the x-ray spot size in the ARPES measurements was about 180 × 80 (H × V) μm^2^, we can assume that we are able to obtain large areas of mono-domain trilayer with Bernal stacking, in contrast to other works, where the epitaxial graphene showed different domains with different numbers of layers or different stacking^10^,^22^. These results make the off-axis SiC(0001) substrate, a good candidate for producing large areas of trilayer graphene with the favorable Bernal stacking.

Finally, to study the electronic quality of our graphene layers, we determined the carrier mobility of the sample using FIR-MT. In order to measure the FIR-MT in the range 0–80 meV, the trilayer graphene sample (typically 5 × 5 mm^2^) was placed in a 15 T superconducting coil at 4 K and exposed to the radiation from a mercury lamp (Faraday geometry). The transmitted light was analyzed by a Fourier transform spectrometer using a Si composite bolometer directly below the sample for detection. The transmission at a given magnetic field T(B) was normalized by the zero field transmission T(0). Typical transmission spectra are shown in Fig. 5(a). For B ≥ 3T, a broad absorption line was mainly observed and attributed to the electron cyclotron resonance of the conduction bands which touch at the K point. Note that the energy position of this minimum did not vary linearly with B, which is the feature of Dirac fermions^23^. The Landau levels (LLs) of an ABA trilayer graphene have been calculated analytically 24,25 and are shown in Fig. 5(b). The trilayer graphene LL consists of both a bilayer graphene-like and a monolayer graphene-like LL spectrum. This is expected since the trilayer graphene band structure consists of one massless monolayer graphene-like band and two massive bilayer graphene-like bands, as discussed above. The energy separation of the bilayer graphene-like LL is too narrow to be experimentally observed (i.e. of the order of the LL broadening so that the different LLs are not resolved) and we essentially measured the monolayer graphene-like spectrum given by 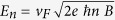, where n is the LL index. The Fermi velocity 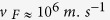
is deduced from the ARPES measurements. The cyclotron resonance corresponds to the transition n→n+1, where the n^th^ level is the upper populated LL (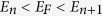), as shown by the arrows in Fig. 5(b). Taking Fermi energy 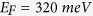 above the Dirac point in agreement with the ARPES measurements, the cyclotron resonance energy can be calculated for each magnetic field. For example, the absorption at B = 15 T associated with the transition 5→6 is indicated by the arrow in Fig. 5(a). By considering that a clear absorption minimum is only defined for B ≥ 3T, one can deduce a mobility of the Dirac fermions higher than 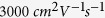 at 4K. The carrier mobility obtained was particularly high and comparable to that which we usually obtained in graphene monolayers^26^. Even if the Bernal stacked trilayer graphene uniformly covers the terraces of the sample creating a very homogenous sample, the presence of the steps edges can effectively increase short-range scattering and affect the mobility^27^. A reduction in step edge density using a gentle hydrogen intercalation between the SiC substrate and the graphene^26^, could probably improve the electron mobility further.

In summary, we have investigated the electronic properties of Bernal stacking trilayer graphene. When large-area of trilayer graphene is measured at low temperature, the CR observation condition μB ≥ 1 allows to deduce an electron mobility of ~3000 cm^2^V^−1^s^−1^. We used NEXAFS to study the lowest unoccupied electronic states, mainly π* and σ*, providing information on the two-dimensional nature of graphene layers on a SiC substrate. By comparing ARPES measurements with band structure calculations, we show that the trilayer graphene presents an ABA stacking. Our approach represents a significant step towards the scalable synthesis of high quality Bernal stacking trilayer on a large scale. It is compatible with the high structural qualities and fine thickness control needed to develop graphene-based electronic devices.

## Methods

The trilayer graphene used in this study was obtained by annealing an off-axis 4H-SiC(0001) substrate. Before graphitization, the substrate was hydrogen etched (100% H2) at 1550 °C to produce well-ordered atomic terraces of SiC. The sample was heated to 820 °C and deoxidized at 1100 °C in order to remove the native oxide and a possible surface contamination. The substrate was then heated to 1550 °C in an Ar atmosphere (800 mbar) for 10 min. The samples were cooled down to room temperature and transferred ex-situ to perform the different measurements.

The AFM measurements were carried out under ambient conditions and the images recorded in tapping mode. The Micro-Raman mapping was performed at room temperature with a Renishaw spectrometer using 532 nm laser light auto-focusing on the sample by a DMLM Leica microscope with a 100× objective and a power of 5 mW with a spot size of about 1 μm.

The XPS, NEXAFS and ARPES experiments were carried out on the TEMPO beamline^28^ (SOLEIL French synchrotron facility) at room temperature^29^. The photon source is a HU80 Apple II undulator set to deliver linear polarized light. During the XPS measurements, the photoelectrons were detected at 0° from the sample surface normal 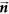 and at 42° from the polarization vector 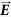. The zero binding energy (BE) (i.e. the FERMI level) was taken at the leading edge of a clean molybdenum foil in electriclal contact with the sample. The C 1s NEXAFS spectra were recorded in the Auger yield mode using an energy window of 13 eV centered at 260 eV. For ARPES measurements, the photon energy (h*ν* = 60 eV) and sample orientation were set in order to explore the k-space region around the K point in the ΓK direction of the Brillouin zone.

## Additional Information

**How to cite this article**: Hajlaoui, M. *et al.* High Electron Mobility in Epitaxial Trilayer Graphene on Off-axis SiC(0001). *Sci. Rep.* **6**, 18791; doi: 10.1038/srep18791 (2016).

## Figures and Tables

**Figure 1:**
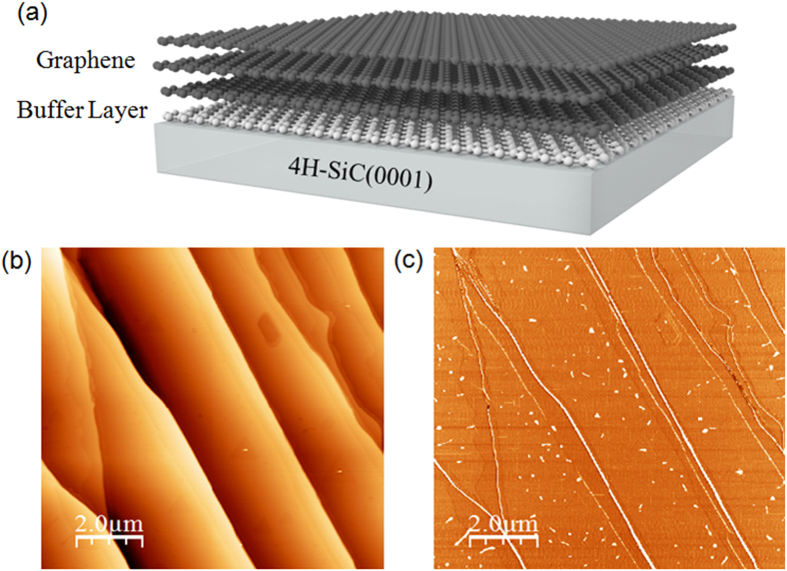
Structural properties of epitaxial trilayer graphene. (**a**) Schematic structure of our trilayer graphene on SiC(0001), (**b**) Morphological AFM image of trilayer graphene on an off-axis SiC(0001) substrate, (**c**) The AFM phase image of trilayer graphene.

**Figure 2:**
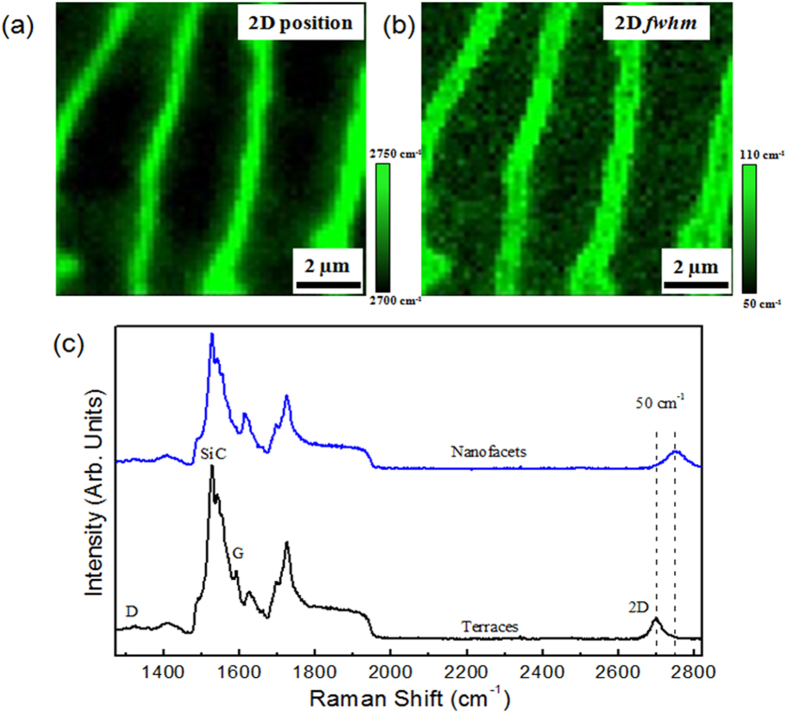
Micro-Raman spectroscopy of epitaxial trilayer graphene. (**a**) and (**b**) show the Raman shift and *fwhm* of the 2D vibrational mode respectively. Image dimensions are 12 *μ*m × 12 *μ*m, (**c**) Micro-Raman spectra of the D, G and 2D vibrational mode collected on the terrace (black spectrum) and on the step edge (blue spectrum), with a 532 nm wavelength excitation and a spatial resolution better than 1 μm.

**Figure 3:**
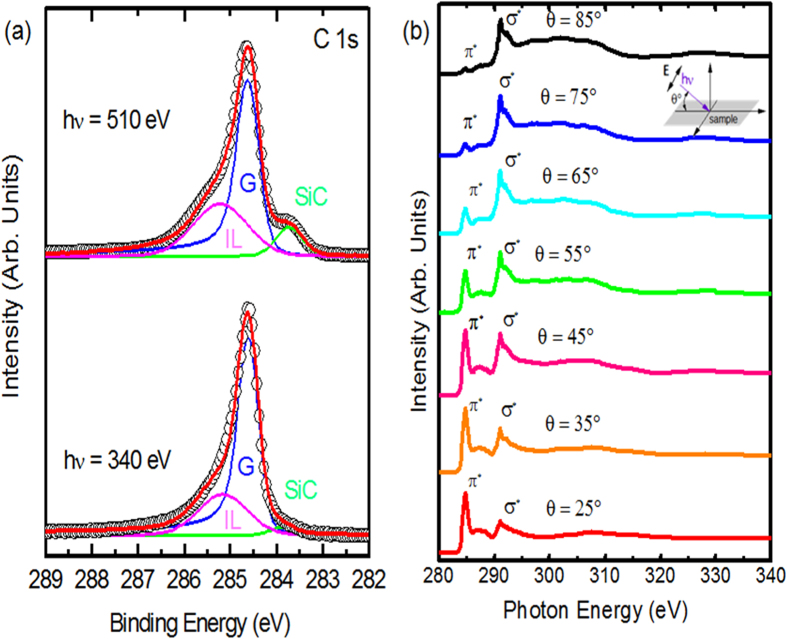
Electronic properties of epitaxial trilayer graphene. (**a**) High resolution XPS spectra of C 1s core level hν = 510 eV (bulk sensitive, top panel) and hν = 340 eV (surface sensitive, bottom panel), (**b**) Carbon K-edge NEXAFS spectra, measured for different incidence angles of linearly polarized synchrotron light. The insert shows a schematic representation of the NEXAFS geometry: θ represents the angle between the incident photons and the sample surface.

**Figure 4:**
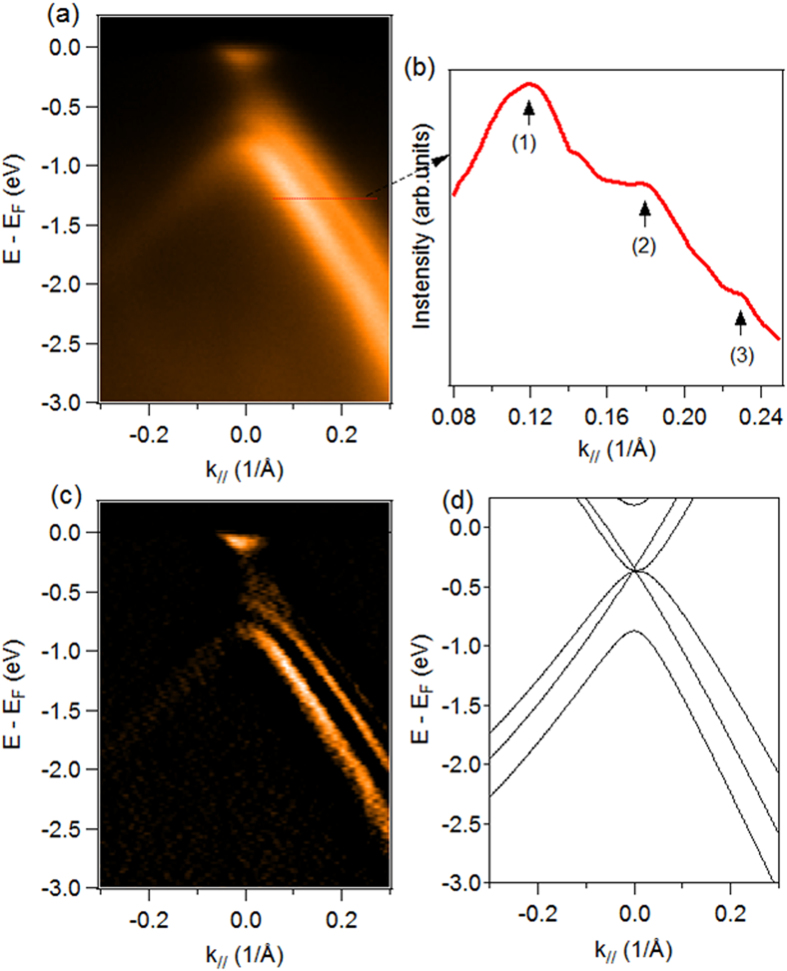
Band structure of epitaxial trilayer graphene. (**a**) ARPES map of the trilayer graphene acquired along the MΓK direction at hν = 60 eV, (**b**) MDC extracted from the ARPES map at E–E_F_ = – 1.32 eV, (**c**) Second derivative of the ARPES map in (**a**,**d**) DFT band structure calculation for Bernal stacking trilayer graphene.

**Figure 5:**
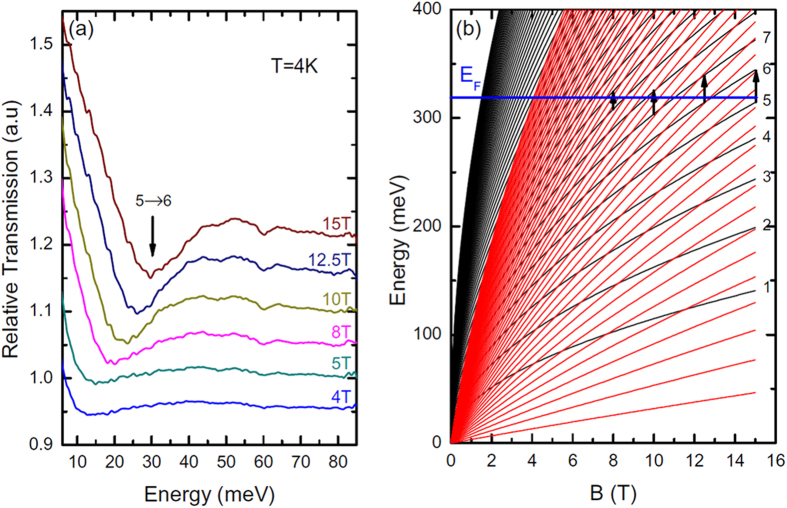
Magneto-transmission measurements of epitaxial trilayer graphene. (**a**) FIR transmission spectra of an ABA trilayer graphene for various magnetic fields B. The arrow shows the calculated energy of the cyclotron resonance 5 → 6 at 15 T. (**b**) Calculated LL spectrum of an ABA trilayer graphene. Monolayer graphene-like and bilayer graphene-like LLs are represented by the black and red lines, respectively. The blue line represents the Fermi energy and the arrows indicate the measured cyclotron resonances between LLs of index n.
